# The Natural Human IgM Antibody PAT-SM6 Induces Apoptosis in Primary Human Multiple Myeloma Cells by Targeting Heat Shock Protein GRP78

**DOI:** 10.1371/journal.pone.0063414

**Published:** 2013-05-07

**Authors:** Leo Rasche, Johannes Duell, Charlotte Morgner, Manik Chatterjee, Frank Hensel, Andreas Rosenwald, Hermann Einsele, Max S. Topp, Stephanie Brändlein

**Affiliations:** 1 Department of Internal Medicine II, University Hospital Würzburg, Würzburg, Germany; 2 Institute of Pathology, University of Würzburg, Würzburg, Germany; 3 Comprehensive Cancer Center Mainfranken, Würzburg, Germany; 4 Patrys GmbH, Würzburg, Germany; Vanderbilt University, United States of America

## Abstract

In contrast to other haematological malignancies, targeted immunotherapy has not entered standard treatment regimens for *de novo* or relapsed multiple myeloma (MM) yet. While a number of IgG-formatted monoclonal antibodies are currently being evaluated in clinical trials in MM, our study aimed to investigate whether the fully human IgM monoclonal antibody PAT-SM6 that targets a tumour-specific variant of the heat shock protein GRP78 might be an attractive candidate for future immunotherapeutic approaches. We here show that GRP78 is stably and consistently expressed on the surface on tumour cells from patients with *de novo*, but also relapsed MM and that binding of PAT-SM6 to MM cells can specifically exert cytotoxic effects on malignant plasma cells, whereas non-malignant cells are not targeted. We demonstrate that the induction of apoptosis and, to a lesser extent, complement dependent cytotoxicity is the main mode of action of PAT-SM6, whereas antibody dependent cellular cytotoxicity does not appear to contribute to the cytotoxic properties of this antibody. Given the favourable safety profile of PAT-SM6 in monkeys, but also in a recent phase I trial in patients with malignant melanoma, our results form the basis for a planned phase I study in patients with relapsed MM.

## Introduction

Multiple myeloma (MM) is a malignant disorder characterized by clonal expansion of plasma cells in the bone marrow leading clinically to anaemia, bone destruction, monoclonal gammopathy, renal failure, hypercalcemia and increased susceptibility to infections [1]. Although treatment options for MM patients have improved over the past decade, MM remains largely incurable. Particularly, relapsed and refractory MM is associated with survival times of less than 30 months [2] resulting in a clear need to develop novel therapeutic approaches across all lines of MM therapy.

Targeted immunotherapy using monoclonal antibodies (mAbs) has substantially improved the treatment of lymphoid malignancies resulting in better initial disease control and corresponding higher rate of remissions in many lymphoma patients [3]. In contrast, antibody-based immunotherapy has not been approved for standard treatment in MM [4] despite several clinical trials testing IgG formatted antibodies. At present, novel candidates including elotuzumab or daratumumab targeting the plasma cell associated antigens CD319 and CD38, respectively, [5], [6] are being explored in phase I/II and III trials, but their success remains to be established [7].

In general, molecules that are selectively overexpressed on the cell surface of neoplastic cells and that play an important role in cell growth or survival represent attractive targets for immunotherapy. Binding of therapeutically effective mAbs exerts cytotoxic effects by directly inducing apoptosis or via complement-dependent cytotoxicity (CDC) or the activation of cytotoxic immune effector functions such as antibody-dependent cellular cytotoxicity (ADCC) [8].

The fully human IgM mAb PAT-SM6 was initially isolated from a patient with gastric cancer and was described to be part of the natural immunity. In a previous study, we could demonstrate that PAT-SM6 targets a tumour specific variant of the heat shock protein GRP78 (BIP, HSPA5) [9]. GRP78 is known to be an endoplasmic reticulum-related protein, but it is also expressed on the cell surface of various epithelial cancer cells [10] and increased expression levels of GRP78 in cancer cells have been correlated with an adverse prognosis and drug resistance [11]. In addition, we and others reported that PAT-SM6 also binds to oxidized low-density lipoprotein (LDL). The simultaneous binding of oxidized LDL and cell-surface GRP78 induces internalisation of oxidised LDL in malignant cells causing intracellular deposition of cholesterol and triglyceride esters which in turn triggers the cells to undergo apoptosis [12], [13].

GRP78 expression data in human tumours, *in vitro* cell-based assays, and *in vivo* human tumour xenograft studies suggest that PAT-SM6 may be an effective anti-tumour agent in several indications. The safety of this antibody treatment was recently demonstrated in cynomolgus monkeys, but also in a phase I trial evaluating safety, pharmacokinetics, immunogenicity, pharmacodynamics and anti-tumour activity of PAT-SM6 in patients with recurrent in-transit cutaneous melanoma (ACTRN 12610000733077).

In this study, we investigate the target expression profile of the IgM antibody PAT-SM6 in primary MM cells and demonstrate anti-tumour effects and their potential underlying mechanisms.

## Materials and Methods

### Ethics Statement

This study was approved by the Ethics Committee of the Medical Faculty of the University of Würzburg (reference no. 44/10) and IRB-approved written informed consent was obtained from all participants.

### Cell Culture

Human multiple myeloma (MM) cell lines INA-6, NCI H929, MM1.S, OPM-2 and U266 were obtained from the German Collection of Microorganisms and Cell Cultures (Braunschweig, Germany), ATCC (Manassas, VA), or kindly provided by M. Gramatzki (Kiel) [14] and maintained as previously described [15]. Primary CD138^+^ MM cells from patients were obtained using positive selection with CD138 microbeads (Miltenyi Biotech, Bergisch Gladbach, Germany).

### Antibodies

Anti-GRP78 antibody PAT-SM6 (fully human IgM) was produced as outlined elsewhere [16] and provided by Patrys Ltd. (Melbourne, Australia). Anti-GRP78 control mAb (rabbit IgG, ET-21) was obtained from Sigma-Aldrich (St. Louis, MO). The anti-CD20 antibody Rituximab (Roche) was used as complement activating control antibody in CDC studies. ChromPure IgM was used as isotype control (Dianova, Germany).

### PAT-SM6 Immunostaining on Bone Marrow Paraffin Sections

Immunohistochemistry (IHC) with PAT-SM6 antibody or control antibodies on bone marrow paraffin sections and cytospin preparations was performed as previously described [17].

### Flow Cytometry

Direct and indirect immunofluorescence flow cytometric analysis was performed using a FACScan with CellQuest Pro acquisition software (Beckman Coulter, Miami, FL). The expression of GRP78 on MM cells was assessed using anti-GRP78 IgG (rabbit), as well as PAT-SM6 followed by fluorescein isothiocyanate (FITC)-conjugated secondary antibodies (Abcam or Dako). Isotype controls (human IgM or rabbit IgG) were used for the assessment of unspecific binding. The expression of CD138 was analysed using anti-CD138-FITC mAb (Beckman Coulter).

The recruitment of C1q to OPM-2 cells facilitated by PAT-SM6 was assessed by incubating cells with C1q (Quidel A400) and PAT-SM6 or IgM isotype control (ChromPure) respectively. Cells were stained using murine anti C1q mAb (Quidel A401) and anti murine IgG conjugated to flourescein (DAKO). Overlay was shown against unspecific C1q binding with IgM isotype control.

### ELISA (Enzyme-linked Immunosorbent Assay)

96-well plates (Corning Costar® 3590, NY) were used for the ELISA experiments. Coating was performed overnight at 4°C with reagents diluted in 0.05 M sodium bicarbonate buffer, pH 9. Samples were prepared as triplicates. Following coating all steps were performed at room temperature. Between incubation steps plates were washed 3 times with PBS/0.05% Tween 20, pH 7.4. Blocking was performed with PBS/0.05% Tween 20/2%BSA, pH 7.4 for 2 h. Tetramethylbenzidine (TMB) substrate was added and the reaction was stopped with 3 M H_2_SO_4_. Absorbance was measured at 450 nm using an ELISA reader.

For complement factor q1 (C1q) binding analysis, plates were coated with antibody concentrations ranging from 0.5 µg/ml to 20 µg/ml using triplicates. After blocking plates were incubated with human C1q (Quidel A400) 2 µg/ml for 2 h followed by sheep anti human C1q-HRP (Abcam, ab 46191) for 2 h.

For IgM binding to recombinant GRP78, plates were coated with GRP78 (produced in HEK293 cells, kindly provided by Patrys GmbH) ranging from 0.1 to 10 µg/ml. After blocking plates were incubated with natural IgM antibodies 2 µg/ml for 3 h followed by incubation with anti-human IgM-HRP (Dako) diluted 3∶5,000 for 2 h.

For competition studies, all wells were coated with 10 µg/ml GRP78. Increasing amounts of GRP78 or control protein with the same molecular weight were added to a solution of PAT-SM6 IgM (2 µg/ml in PBS/0.05% Tween 20/0.2%BSA, pH 7.4). After incubation for 3 h anti-human IgM-HRP (Dako) diluted 3∶5,000 was added and incubated for 2 h.

### Cytotoxicity Assays

MM cell lines (2×10^5^ cells/ml) or CD138-purified patient MM cells were incubated with PAT-SM6 or human isotype control IgM (0–400 µg/mL) in 96-well plates for 48 h (primary MM cells) or 72 h (MM cell lines). MM cell lines were tested in RPMI containing 1% FCS. Primary MM cells were tested in RPMI containing 10% FCS and IL-6 (2,5 ng/mL).

Cell death was determined by FACS analysis using the guava via count kit (Millipore Merck, Darmstadt, Germany). Propidium iodide and LDS-75 double positive cells were set as dead cells. Annexin and 7-AAD positive cells were determined using the Nexin Kit (Milipore Merck) according to manufacturer’s protocol.

### Caspase 3 Activation Assay using PAT-SM6 in the MM1.S Cell Line

Determination of caspase 3 activation was performed using the AC-DEVD-AMC protease assay (BD Biosciences Pharmingen) as previously described by Mashima et al. [18]. Briefly, 2×10^5^ cells were incubated with PAT-SM6 or controls (isotype as negative control or staurophorin as positive control) in a 6-well plate in a total volume of 2 mL/well. After incubation for 3 h, 6 h, 24 or 48 h, respectively, cells were harvested, centrifuged at 13,000 rpm and stored at −20°C. Cell pellets were lysed (200 mM Tris pH 7.5, 2 M NaCl, 20 mM EDTA, 0.2% Triton X 100 lysis buffer), centrifuged, and supernatants were transferred to a 96-well plate. Reaction buffer (50 mM Pipes pH 7.4, 10 mM EDTA, 0.5% Chaps) was added to the wells with or without caspase 3 inhibitor (AG Scientific, San Diego, CA) and incubated overnight at room temperature. RFU counts were determined using a fluorescent reader.

### CDC Assays

50 µl of cell suspension (10,000 cells per well) was added into each well of a 96-well plate followed by 50 µl of antibody dilution (starting from 0.01 µg/ml up to 400 µg/mL total concentration/well diluted in RPMI containing 1% FCS) of PAT-SM6, rituximab or controls. After incubation for 15 min, 50 µL complement (normal human complement, Quidel, San Diego, CA) was added. The plates were incubated for 2 hours at 37°C with 5% CO_2_. After incubation the plates were centrifuged at 2,000 rpm for 5 min and supernatant was discarded. 30 µl of PBS buffer and 270 µl of a prepared solution of propidium iodide and LDS75 (Via Count Reagent, Millipore) were added to each well. After incubation for 15 minutes in the dark, cell viability was examined by micro-capillary flow cytometry (Guava PCM96 platform) and the ratio of live to dead cells was calculated for each well by counting 2,000 events per well using Cytosoft software. All experiments were repeated at least three times.

CDC activity was assessed by alamar blue as described previously [19]. Briefly, 50 µl cell suspension (10,000 cells) was transferred into each well followed by 50 µl dilution of antibody PAT-SM6 or rituximab (starting from 1 µg/ml up to 400 µg/mL total concentration/well) and isotype control (Chrompure IgM 400 µg/mL total concentration/well). To set a control for 100% lysis a Triton-X100 solution (2.5 Vol%) was used. After incubation at 37°C and 5% CO_2_ for 15 min, 50 µl complement was added to each well, followed by 15 µl of alamar blue solution (10 µl/100 µl cell suspension according to manufacturer) after 2 h incubation. All dilutions were performed in RPMI containing 10% FCS. Plates were incubated for 16 hours at 37°C and 5% CO_2_ and subsequently analysed using a fluorescence reader. All experiments were repeated at least three times.

### ADCC Assay

CFSE labelled MM cell line OPM-2 was incubated with PAT-SM6 (20 µg/mL or 50 µg/mL) or unrelated polyclonal IgM (ChromPure, Dianova, Germany). Peripheral blood mononuclear cells (PBMC) from healthy donors in an effector to target ratio of 25∶1 or 50∶1 in RPMI supplemented with 1% human serum were added. After incubation of 4 or 24 h cells were stained with 7-AAD for cell death determination and analysed by FACS. 7-AAD/CFSE double positive cells were set as MM cells killed by ADCC.

### Antibody Induced NK Cell Activation Assessed by CD69 Expression

OPM-2 MM cells were incubated with PAT-SM6 or unrelated polyclonal IgM (ChromPure, Dianova, Germany) for 15 min on ice followed by centrifugation. Cells were transferred into a 24-well plate (flat bottom) and co-incubated with PBMC from a healthy donor in an effector to target ratio of 10∶1 for 0 and 20 hours. NK cell activation was determined using CD56, CD3 and CD69 fluorescent conjugated antibodies (BD, Germany) analysed by FACS.

### Statistical Analysis

*In vitro* experiments were repeated in triplicates, and the results are reported as mean with standard error. Statistical significance of differences observed in experimental versus control condition was determined using the Student t-test or Wilkoxen sum rank test. The minimal level of significance was P<0.05. The statistical analysis was performed using GraphPad prism 5.

## Results

### PAT-SM6 Specifically Binds to Primary MM Cells

**Figure 1:**
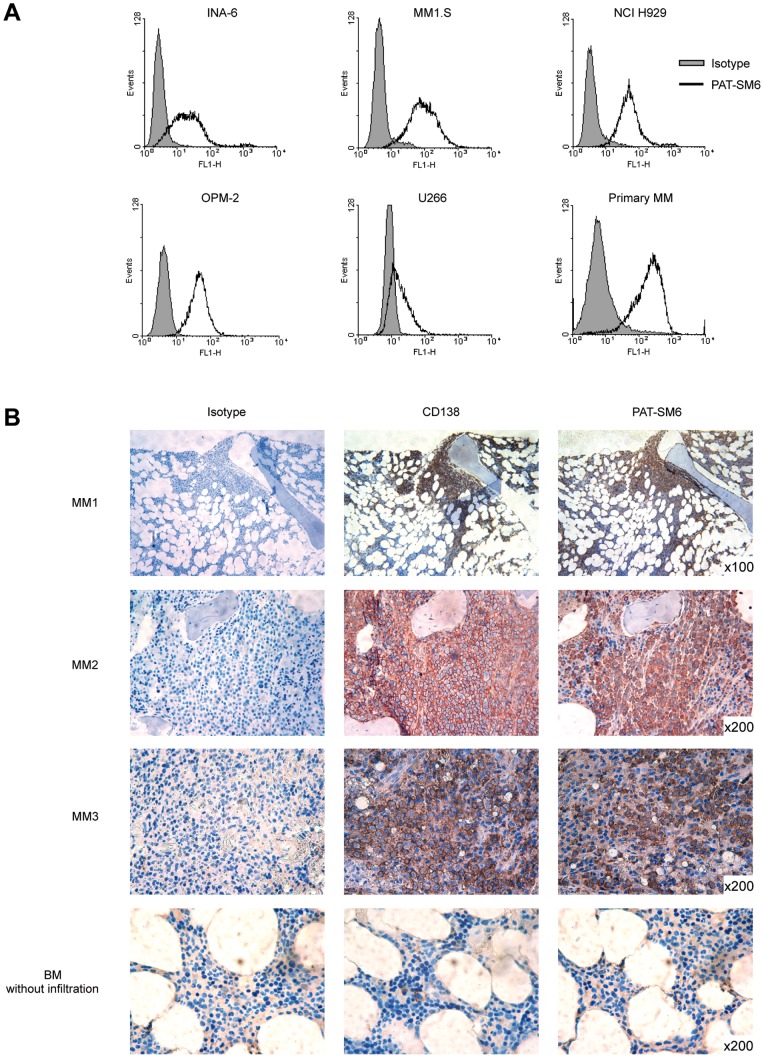
PAT-SM6 specifically binds to MM cell lines and primary human MM samples. A, INA-6, NCI H929, MM1.S, OPM-2 and U266 MM cell lines or CD138-purified patient MM cells were stained with PAT-SM6 (5 µg/mL, open histogram) or isotype control (gray histogram) followed by fluorescein isothiocyanate (FITC)-conjugated secondary antibodies and assessed by flow cytometry. Both, cell lines as well as primary MM cells displayed specific PAT-SM6 binding. B, Immunohistochemistry with PAT-SM6, CD138-positive control antibody or isotype on bone marrow biopsies of MM patients. PAT-SM6 specifically stained MM cells with a homogenous binding pattern. In samples without MM infiltration no specific binding was detected. Images were captured using a Leica DM BL microscope, the Leica ICC HD digital camera and the Leica LAS EZ V2.1.0 software.

A panel of 10 monoclonal patient-derived IgM antibodies was screened for their binding capacity to MM cells. All selected IgM antibodies had shown tumour reactivity with a broader panel of different carcinomas in previous studies [17]. The human IgM antibody PAT-SM6 showed strongest staining by immunohistochemistry (IHC) on cytospin preparations and displayed binding to all tested MM cell lines (INA-6, NCI H929, MM1.S, OPM-2 and U266) in FACS analysis (Fig. 1A). Therefore, PAT-SM6 was selected for further analysis in primary human MM cells. Here, PAT-SM6 specifically reacted with CD138-purified cells from MM patients displaying 100% binding by FACS (Fig. 1A). Furthermore, a homogenous membrane staining could be observed in cytospin preparations, whereas CD138+ normal plasma cells which were obtained from healthy volunteers showed no PAT-SM6 binding, indicating a tumour cell specific binding (Figure S1).

### PAT-SM6 Binds to MM Cells in Bone Marrow Infiltrates in All Cases

To generate representative data on the expression pattern of the PAT-SM6 target structure in MM patients, immunohistochemical staining in bone marrow biopsies was performed. In total, 24 samples were evaluated, including 11 MM patients at primary diagnosis, 9 MM patients in first or later relapse (Table 1) and 4 samples from healthy donors. PAT-SM6 specifically bound to MM cells in all 20 samples with a homogenous staining pattern of 80–100% of tumour cells (mean: 92%), indicating that its target structure is stably expressed in MM throughout various stages of the disease. A statistically significant difference in the percentage of PAT-SM6 positively stained cells MM cells at primary diagnosis vs. relapse could be observed (87% vs. 97%, p = 0.01, Table 1). In 8 of 20 samples, staining was limited to MM cells and did not show any background reaction (Fig. 1B). In 12 samples very minor background staining of less than 4% of nucleated cells was observed that affected mainly myeloid progenitors. The four healthy controls showed no PAT-SM6 binding reaction (representative example shown in Figure 1B).

**Table 1:** Immunohistochemical analysis of the PAT-SM6 target expression pattern in bone marrow biopsies of MM patients.

Primary Diagnosis			
Pat. Nr.	MM Infiltration (CD138+)	SM-6 stained CD138+ MM cells	SM-6 stained cells other than myeloma
1	60%	90%	myelopoiesis (5%)
2	25–30%	100%	myelopoiesis (5%)
3	50–70%	80–90%	myelopoiesis (5%)
4	40%	90%	myelopoiesis (10%)
5	20–30%	80–90%	myelopoiesis (5%)
6	30–40%	80%	myelopoiesis (5%)
7	80–90%	80%	none
8	70%	100%	none
9	70–80%	80–90%	none
10	60–70%	90%	myelopoiesis (10%)
11	85%	80%	none
**total**		**87%**(range 80–100%)	4%(range 0–10%)
**Relapse**			
**Pat. Nr.**	**MM Infiltration (CD138+)**	**PAT-SM6 stained CD138+ MM cells**	**SM-6 stained cells other than myeloma**
1	70%	80%	myelopoiesis (5%)
2	30%	100%	none
3	20%	90%	none
4	70–80%	100%	none
5	40–50%	100%	none
6	25–30%	100%	myelopoiesis (10%)
7	70%	100%	myelopoiesis (5%)
8	50%	100%	myelopoiesis (5%)
9	20%–30%	100%	myelopoiesis (5%)
**total**		**97%**(range 80–100%)	3,3%(range 0–10%)

### GRP78 Target Confirmation by ELISA, Immunohistochemistry and FACS

We have previously shown that PAT-SM6 binds to a tumour-specific isoform of GRP78, a member of the HSP70 family, as well as to oxidized LDL [9], [17]. Binding of PAT-SM6 to GRP78 and oxidized LDL induces a tumour cell specific apoptotic process, called lipoptosis [12]. Given the broad reactivity pattern of PAT-SM6 we first investigated whether GRP78 is also the target structure of this antibody on MM cells.

**Figure 2:**
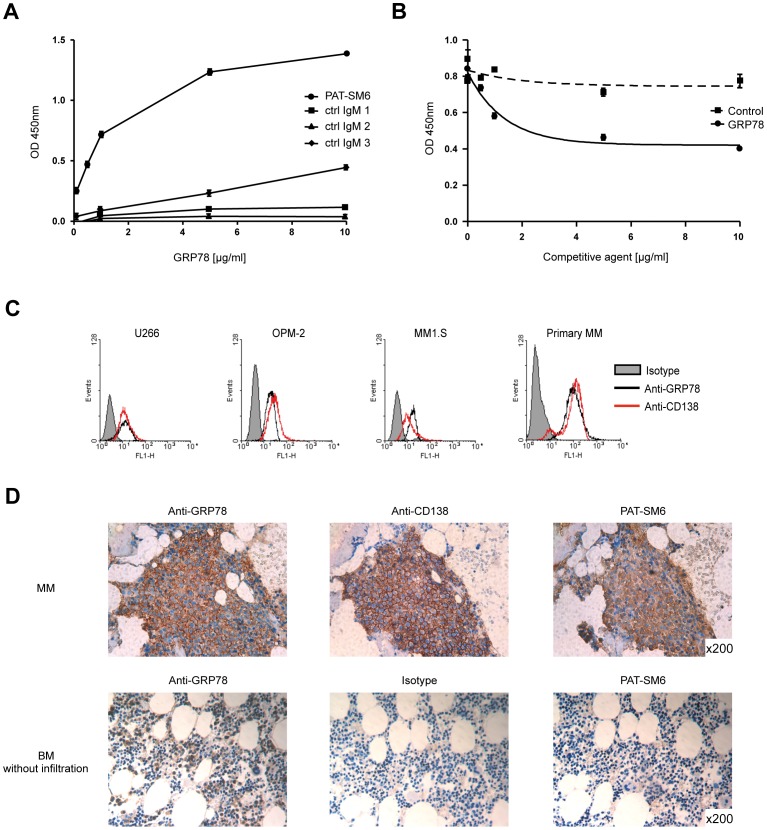
Target validation, PAT-SM6 reacts with cell surface expressed GRP78. A, Sandwich ELISA with recombinant GRP78 and PAT-SM6. Plates were coated with GRP78, blocked and incubated with PAT-SM6 or other natural IgM antibodies as controls followed by incubation with anti-human IgM-HRP. PAT-SM6 showed binding to recombinant GRP78. B, Competitive ELISA: Plates were coated with GRP78 and increasing amounts of GRP78 or control protein were added to a solution of PAT-SM6 followed by anti-human IgM-HRP. Unbound GRP78 reduced binding of PAT-SM6 on a GRP78 coated plate. C, Surface expression GRP78 (black histogram) in MM cell lines is shown using an anti GRP78 IgG (rabbit) antibody. D, GRP78 expression in bone marrow biopsies was assessed by immunohistochemistry with anti GRP78 IgG, PAT-SM6 or positive control CD138. Primary MM cells were positive for GRP78 and the staining pattern correlates with the pattern displayed by PAT-SM6. In contrast to PAT-SM6, anti-GRP78 IgG also reacted with non-malignant cells. Images were captured using a Leica DM BL microscope, the Leica ICC HD digital camera and the Leica LAS EZ V2.1.0 software.

In ELISA binding studies, PAT-SM6 bound to recombinant GRP78 in a dose dependent manner, whereas other unrelated natural monoclonal IgM antibodies showed no reaction (Fig. 2A). Findings were further validated in a competitive ELISA assay, in which unbound GRP78 reduced binding of PAT-SM6 on a GRP78 coated plate. A control protein with similar molecular weight showed no effect. Thus, GRP78 is the target structure of PAT-SM6 (Fig. 2B).

We next analysed the expression pattern of GRP78 in MM cell lines and primary MM cells by FACS and immunohistochemistry (IHC) using a rabbit anti-GRP78 IgG antibody. GRP78 was expressed in all MM cell lines and primary MM cells tested. FACS analysis revealed the target to be expressed on the plasma membrane of the cells (Fig. 2C). IHC staining of bone marrow sections indicated that MM cells stained positive for wild-type GRP78, and that the staining pattern correlated with the pattern displayed by PAT-SM6 (Fig. 2D). However, in contrast to PAT-SM6, the rabbit anti-GRP78 IgG antibody reacted with non-malignant cells as well. These findings provide additional evidence that PAT-SM6 targets a tumour specific variant of GRP78 [9]. Taken together, the PAT-SM6 specific GRP78 variant is widely expressed on the cell surface of MM cells, but not on healthy cells.

### PAT-SM6 Mediates Cytotoxicity to MM Cells by Induction of Apoptosis and Complement Activation

**Figure 3:**
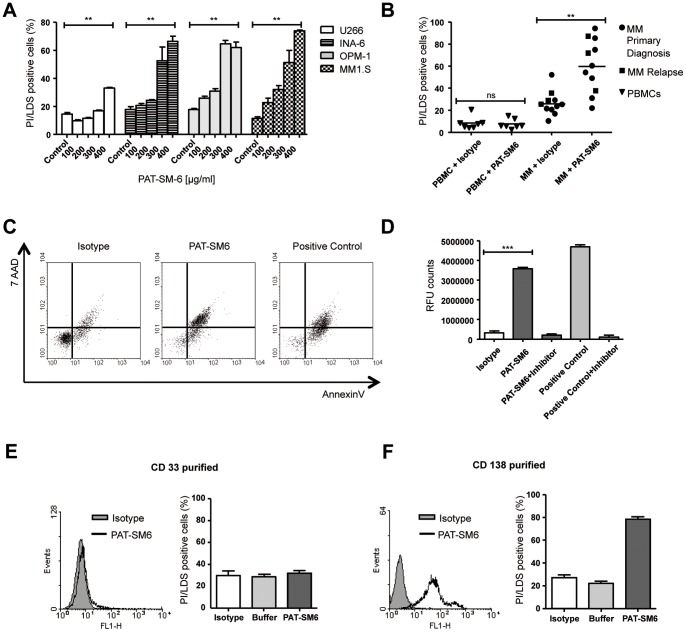
PAT-SM6 mediates cytotoxicity to patient MM cells and MM cell lines by induction of apoptosis. A, MM cell lines were incubated with various concentrations of PAT-SM6 or control (unrelated IgM at highest concentration) for 72 h. Cell death was determined by FACS (Propidium iodide/LDS75 double positive cells). B, CD138-purified primary MM cells at primary diagnosis (n = 8, dots) or relapse (n = 3, rectangle) were incubated for 48 h with PAT-SM6 or isotype in media containing IL-6 and 10% FCS. Dead cells were determined by FACS analysis (Propidium iodide/LDS75 double positive cells). As control, PBMCs from healthy volunteers were treated under the same conditions. In contrast to controls PAT-SM6 significantly induced programmed cell death. C, MM1.S cells were incubated with PAT-SM6 or controls (staurophorin as positive control) for 72 h. AnnexinV and 7AAD positive cells were assessed by flow cytometry. Percentage of 7-AAD/Annexin V double positive cells, Isotype: 14%, PAT-SM6∶79% and positive control: 76%. D, MM1.S cells were incubated with PAT-SM6 or controls (staurophorin or isotype) for 6 h and caspase 3 activation was measured using the AC-DEVD-AMC protease assay. E, Bone marrow cells from a MM patient were CD33-purified to obtain myeloid progenitors and stimulated with PAT-SM6 or controls. No specific binding and subsequently no specific cytotoxicity was observed. F, Cells from the same patient as in E were CD138-isolated and incubated with PAT-SM6 (200 µg/mL) for 24 h. Specific PAT-SM6 binding and killing was observed.

We previously demonstrated that PAT-SM6 induces apoptosis after cell surface binding to GRP78 followed by internalization into tumour cells [13]. To elucidate the ability of PAT-SM6 to induce apoptosis in MM cells as well, MM cell lines and primary MM cells were analysed by a propidium iodide/LDS75 based FACS assay after treatment with PAT-SM6. MM1.S, OPM-2, INA-6 and U266 cells displayed a dose dependent cell death when incubated with PAT-SM6. At the highest concentration, PAT-SM6 significantly increased the percentage of dead cells (median range: 33–74.3%, Fig. 3A). Interestingly, U266 cells showed the lowest antibody induced cytotoxic effect, in line with only modest binding of PAT-SM6 to these cells in FACS analysis (Fig. 1A).

We next sought to validate the apoptotic effect of PAT-SM6 in CD138-purified primary MM cells that were freshly obtained from MM patients (n = 11) and cultivated in the presence of IL-6. PAT-SM6 showed an induction of apoptosis in MM cells (mean: 58.2%; median range: 19.2–94.6%, Fig. 3B). Of note, samples from patients at initial diagnosis of MM (n = 8) and samples from relapsed patients (n = 3) showed no significant difference (p = 0.7) with mean apoptotic rates of 56.5% and 62.7%, respectively. We conclude that PAT-SM6 exerts apoptotic effects in MM cells from patients with *de novo* MM as well as in cells from patients with several relapses.

In contrast, PBMCs isolated from blood of healthy donors showed no cytotoxicity when treated with PAT-SM6 (Fig. 3B) confirming the tumour specific cell death induced by PAT-SM6.

Cytotoxicity induced by PAT-SM6 was also observed in MM cell lines by AnnexinV/7-AAD staining and an increased number of AnnexinV/7-AAD double stained cells in primary MM cells (Fig. 3C). Finally, induction of apoptosis was proven by a caspase 3 activation assay in MM1.S cells, indicating antibody mediated apoptosis as the putative mode of action (Fig. 3D).

Since we had observed a minor background staining of PAT-SM6 in myeloid progenitor cells in some bone marrow sections by immunohistochemistry (Table 1), we wished to evaluate the specificity in more detail. We thus isolated CD33-positive myeloid progenitor cells from the bone marrow of a MM patient and performed binding and cytotoxicity studies as described above. PAT-SM6 neither showed reactivity with CD33-positive cells nor induction of any specific cytotoxic effect (Fig. 3E) suggesting an artificial background staining in immunohistochemical analyses. In contrast, CD138-positive MM cells from the same patient showed PAT-SM6 binding in FACS analysis and significant induction of cytotoxicity (Fig. 3F), again demonstrating the specific induction of cytotoxic effects by PAT-SM6.

#### Complement dependent cytotoxicity (CDC)

**Figure 4:**
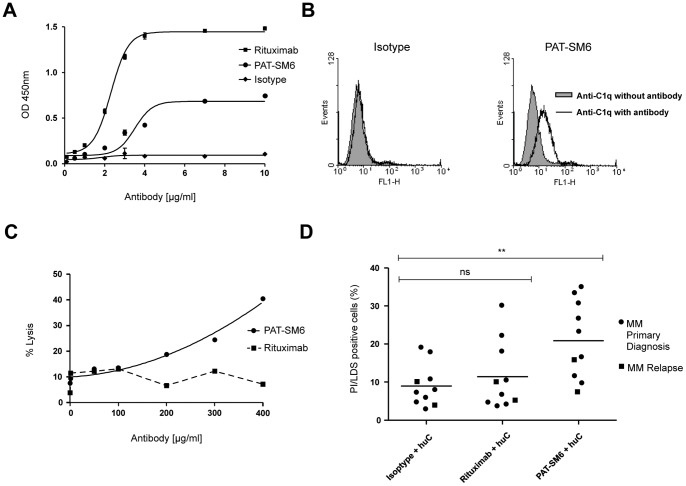
PAT-SM6 binds to C1q and mediates complement deposition and activation on MM cell lines and patients material. A, Sandwich ELISA: For C1q binding analysis, plates were coated with PAT-SM6 or controls (isotype or Rituximab). After blocking, plates were incubated with human C1q followed by sheep anti human C1q-HRP. PAT-SM6 showed clear but moderate binding to C1q compared to the isotype control. Rituximab displayed a stronger C1q binding capacity. B, PAT-SM6 mediated deposition of C1q on the surface of OPM-2 cells assessed by using human C1q and detecting antibodies in FACS. C1q deposition was clearly improved with PAT-SM6 compared to cells without antibody treatment or isotype control. C, CDC activity was determined using the alamar blue assay. OPM-2 cells were incubated with human complement (huC), PAT-SM6 or controls for 2 hours followed by alamar blue solution and the amount of lysed cells was analysed with a fluorescence reader. A dose dependent killing kinetic was observed. D, CD138-purified primary MM cells at primary diagnosis (n = 8, dots) or relapse (n = 2, rectangle) were incubated with human complement, PAT-SM6 or controls (isotype, Rituximab) for 2 h and the amount of lysed was assessed by FACS (Propidium iodide/LDS75). In the PAT-SM6 treated samples a moderate killing was observed, whereas Rituximab showed no significant cytotoxicity.

Although IgM antibodies are described to be excellent complement binders, there is still conflicting data whether IgM antibodies can exert cytotoxic effects by complement dependent cytotoxicity. We therefore determined the capability of PAT-SM6 to bind and activate complement. PAT-SM6 showed clear, but moderate binding to C1q in an ELISA format compared to an unspecific IgM isotype control. However, the IgG antibody Rituximab serving as a positive control, displayed a stronger C1q binding capacity (Fig. 4A), which correlates with superior C1q binding properties of IgG antibodies in general.

To assess complement binding in a living cell system, PAT-SM6-mediated deposition of C1q on the surface of OPM-2 cells was assessed by FACS using human C1q and corresponding antibodies. C1q deposition was clearly improved with the addition of PAT-SM6 compared to cells treated with the isotype control (Fig. 4B).

In order to determine complement-dependent cytotoxicity, OPM-2 cells were co-incubated with various concentrations of PAT-SM6 and human complement for 2 h, followed by the measurement of lysed cells. PAT-SM6 treated cells showed a dose-dependent cell death with a maximum of 40% lysed cells. Due to the lack of CD20 expression in OPM-2 cells, Rituximab in the same concentrations displayed no specific activity (Fig. 4C). Finally, CDC activity was validated in primary MM cells (n = 10). PAT-SM6 induced significant CDC with a rate of 20.9% (range 6.9–35%) vs. 11.4% (range 3.6–30%)(Fig. 4D).

#### Antibody-dependent cellular cytotoxicity (ADCC)

While there is good evidence that some therapeutic IgG antibodies mediate antitumoural cytotoxicity via ADCC [5], [20] the role of ADCC in the cytotoxic properties of IgM antibodies is rather unclear. We therefore analysed the ADCC activity of PAT-SM6 in CFSE labelled target cells (OPM-2), PBMCs or purified natural killer (NK) cells as effectors in a FACS-based assay. No specific cell-mediated lysis of those target cells was observed (Figure S2).

To investigate putative immunomodulatory effects of IgM antibodies, we investigated CD69 and CD25 expression on effector subsets (T-, B- and NK cells) as surrogate markers for an early activation phenotype or as a marker indicating inhibition of immune effector cells. Whereas T- and B-cells showed no significant expression changes, we confirmed findings by Pricop and colleagues showing that unrelated polyclonal human IgM can inhibit NK cell activation [21]. In contrast to human polyclonal IgM, the monoclonal PAT-SM6 antibody did not change the activation marker CD69 on NK cells indicating diverse biological features of human monoclonal IgMs (Figure S3).

## Discussion

Although novel therapeutic agents, such as thalidomide, lenalidomide and the proteasome inhibitor bortezomib have advanced treatment options for MM patients resulting in prolonged survival times [22], [23], MM nevertheless still remains largely incurable and patients tend to relapse even after highly intensified treatment approaches [23].

While antibody-based therapies are widely employed in hematological malignancies, they have not yet entered standard treatment in MM. This may be due, at least in part, to the lack of optimal targets. However, several novel mAbs have been developed during the last years, including monoclonal IgG antibodies or related immune conjugates that specifically target CD38, CS1, CD40, CD74, CD70, HM1.24, interleukin-6 and β2-microglobulin (β2M) [8]. A panel of these IgG formatted antibodies are currently evaluated in phase I to III studies [7]. Most of the initial observations in MM suggest that these mAbs given as mono-therapy have limited antitumor activity; however, a combination approach with other anti-myeloma agents may be a promising new treatment strategy in myeloma.

We here show that the IgM antibody PAT-SM6 targets the heat shock protein GRP78, which is stably and consistently expressed on the surface of primary multiple myeloma cells from patients with *de novo* MM, but also from patients with multiple relapses. Binding of PAT-SM6 to the surface of MM cells results in the induction of apoptosis and CDC, but not ADCC, demonstrating that a fully human IgM antibody can specifically target and eliminate malignant plasma cells.

Natural IgM antibodies are produced from germ line immunoglobulin genes with few, if any, mutations. As a consequence of the low mutation frequency, natural IgMs have low binding affinity and a restricted binding repertoire. However, the multi-valency of the pentameric structure creates high avidity and oligo-reactivity to conserved structures (e.g. carbohydrates, phospholipids, nucleic acids, and heat shock proteins), which makes them promising therapeutic candidates. A single IgM antibody is able to bind multiple copies of its specific target on a tumour cell, leading to effective cross-linking of the targets and efficient induction of cell death.

Several monoclonal IgM antibodies have been developed as therapeutic agents and are administered in clinical trials where evidence of efficacy was gathered [24]–[27]. Despite the growing interest and clinical promise, however, IgM antibodies have so far failed to gain widespread commercial interest as these antibodies are considered to be difficult to handle in terms of production and purification. Due to the large size and the extensively glycosylated nature of these proteins, the production of sufficient quantities of functionally active recombinant IgMs is indeed challenging. However, over the last years, the availability of human therapeutic IgM in GMP-grade is becoming more feasible due to the implementation of recombinant expression systems like Per.C6® [28] and successful increase in fermentation scale [16] making IgM generation economically competitive to IgG antibodies. The Per.C6® cell derived IgM antibody PAT-SM6 proved to be safe in toxicity, toxicokinetics, and immunogenicity studies in cynomolgus monkeys, showing no adverse reactions. Moreover, data generated in humans from a phase I study in patients with recurrent in-transit cutaneous melanoma, demonstrated a good safety profile without any reported serious adverse events. In post-treatment biopsies it could be demonstrated that the melanoma cells are indeed reached and targeted by this IgM antibody *in vivo* (data not shown).

The results of our study suggest that the induction of programmed cell death (PCD) is the major effector mechanism of cell death in MM cells targeted by PAT-SM6. This finding is in line with previous observations in epithelial cancer types. The induction of apoptotic processes is a common feature of natural IgM antibodies as part of the immunosurveillance mechanisms against malignant cells [17]. PAT-SM6 also activates CDC in MM cells by recruiting the complement factor C1q. Compared to the induction of programmed cell death, complement dependent cytotoxicity appears to be moderate, but may represent an additive effect to the main effector mechanism. A possible explanation for this moderate CDC activity of PAT-SM6 could be that pentameric natural human antibodies, which can pass epithelial barriers via the polymeric immunoglobulin receptor, try to avoid damage to healthy tissues, e.g. by complement activation in the intestine or the lungs [29]. PAT-SM6, however, does not induce ADCC, in line with previously reported results of other IgM antibodies [30]. Thus, a combination therapy of PAT-SM6 with other anti-myeloma agents that induce ADCC, e.g. lenalidomide or proteasome inhibitors, might exert synergistic effects in inducing myeloma cell death [7].

We have previously shown that PAT-SM6 cross-reacts with both cell surface expressed GRP78 and oxidized LDL thereby inducing apoptosis in target cells [9], [12], [13]. GRP78 belongs to the group of heat shock proteins (HSP) which have attracted attention in MM in recent years, as their dysregulation may contribute to the pathogenesis of MM [31]–[33]. HSPs are ubiquitous molecular chaperones involved in posttranslational folding, stabilization, maturation and activation of various proteins that are essential mediators of signal transduction and cell cycle progression. Environmental and other stress inducing stimuli lead to an increased intracellular synthesis of HSPs [34]. At high levels, the chaperones protect the cell against ER stress-induced apoptosis and maintain cell viability after exposure to stress-inducing conditions [35]. The result is a complex network of regulators and protective mechanisms, which help the cell to respond to stress stimuli [36] and to prevent cellular degradation processes, induced by apoptosis and autophagocytosis. HSPs are normally cytoplasmic proteins, but under diverse pathological conditions they are over-expressed and found on the surface of cells where they may serve as cell-surface signalling receptors [37]. As in malignant cells hypoxia and glucose deprivation is considered to be a permanent matter of stress, it is not surprising that tumour cells up-regulate HSPs to prevent cellular and molecular damage [11], [38], [39].

GRP78 (BIP, HSPA5) is a member of the HSP70 family and cell-surface-located GRP78 is found in a variety of malignant tumours including breast, lung, gastric, hepatocellular and prostate cancers. The expression correlates with tumour progression, metastasis formation and drug resistance [38]. Of note, natural antibodies to GRP78 were found in the serum of prostate cancer patients, which further points to the antigenic property of surface GRP78 [40], [41].

In summary, we show that the PAT-SM6 target GRP78 is widely expressed on the surface of primary MM cells, but not on normal plasma cells or other hematopoietic or non-hematopoietic cells. Binding of PAT-SM6 to the surface of MM cells, results in the effective induction of apoptosis and CDC, thus making this natural IgM antibody an attractive novel agent in the armamentarium of treatments for MM patients. As a fully human antibody, PAT-SM6 may reduce the risk of host-protective immune responses. The favourable toxicity profiles of PAT-SM6 combined with its high avidity binding to the target GRP78 and its interaction with host immune effectors form the basis for a phase I multi-dose escalating study in patients with relapsed multiple myeloma. This trial commenced at the end of 2012 and is currently ongoing.

## Supporting Information

Figure S1 **PAT-SM6 displays a membrane staining in cytospin preparations of patients MM cells.** CD138-purified primary MM cells were centrifuged by cytospin, fixed and permeabilised with acetone and incubated with PAT-SM6 or controls (isotype, anti CD138). Detection followed with corresponding HRP-conjugated secondary antibodies. PAT-SM6 showed binding to CD138 purified primary MM cells specifically. As non-malignant control, plasma cells were obtained from 50 mL peripheral blood from healthy volunteers by Ficoll gradient centrifugation and subsequently CD138 isolation using magnetic beads. CD138 isolated cells from a healthy donor showed no binding when incubated with PAT-SM6. Images were captured using a Leica DM BL microscope, the Leica ICC HD digital camera and the Leica LAS EZ V2.1.0 software.
(TIF)

Figure S2 **PAT-SM6 does not induce ADCC on OPM-2 cells.** CFSE labelled MM cells were incubated with PAT-SM6 (25 µg/mL or 50 µg/mL) or controls (polyclonal IgM) and PBMC from healthy donors in an effector to target ratio of 25∶1 in RPMI supplemented with 1% human serum. 4 or 24 h after incubation cells were stained with 7-AAD for cell death determination and analysed by FACS. 7-AAD/CFSE double positive cells were set as MM cells killed by ADCC.
(TIF)

Figure S3 **In contrast to polyclonal IgM PAT-SM6 shows no impairment in NK cell activation assessed by CD69 expression.** OPM-2 myeloma cells were incubated with PAT-SM6 or unrelated polyclonal IgM (Chrompure, Dianova, Hamburg, Germany) for 15 min on ice followed by centrifugation and dismissing of supernatant to relieve unbound antibody. Cells were transferred into a 24 well plate (flat bottom) and co-incubated with PBMC from a healthy donor in an effector to target ratio of 10∶1 for 0 (black histogram) and 20 hours (blue histogram). NK cell activation was determined using CD56, CD3 and CD69 fluorescent conjugated antibodies (BD, Heidelberg, Germany). Whereas polyclonal IgM inhibits NK cell activation PAT-SM6 showed no inhibitory activity.
(TIF)
