# Supplementary figures and images for: The Natural Human IgM Antibody PAT-SM6 Induces Apoptosis in Primary Human Multiple Myeloma Cells by Targeting Heat Shock Protein GRP78
Source: PLoS One. 2013 May 7;8(5):e63414. doi: 10.1371/journal.pone.0063414 (PMC3646784; doi:10.1371/journal.pone.0063414)

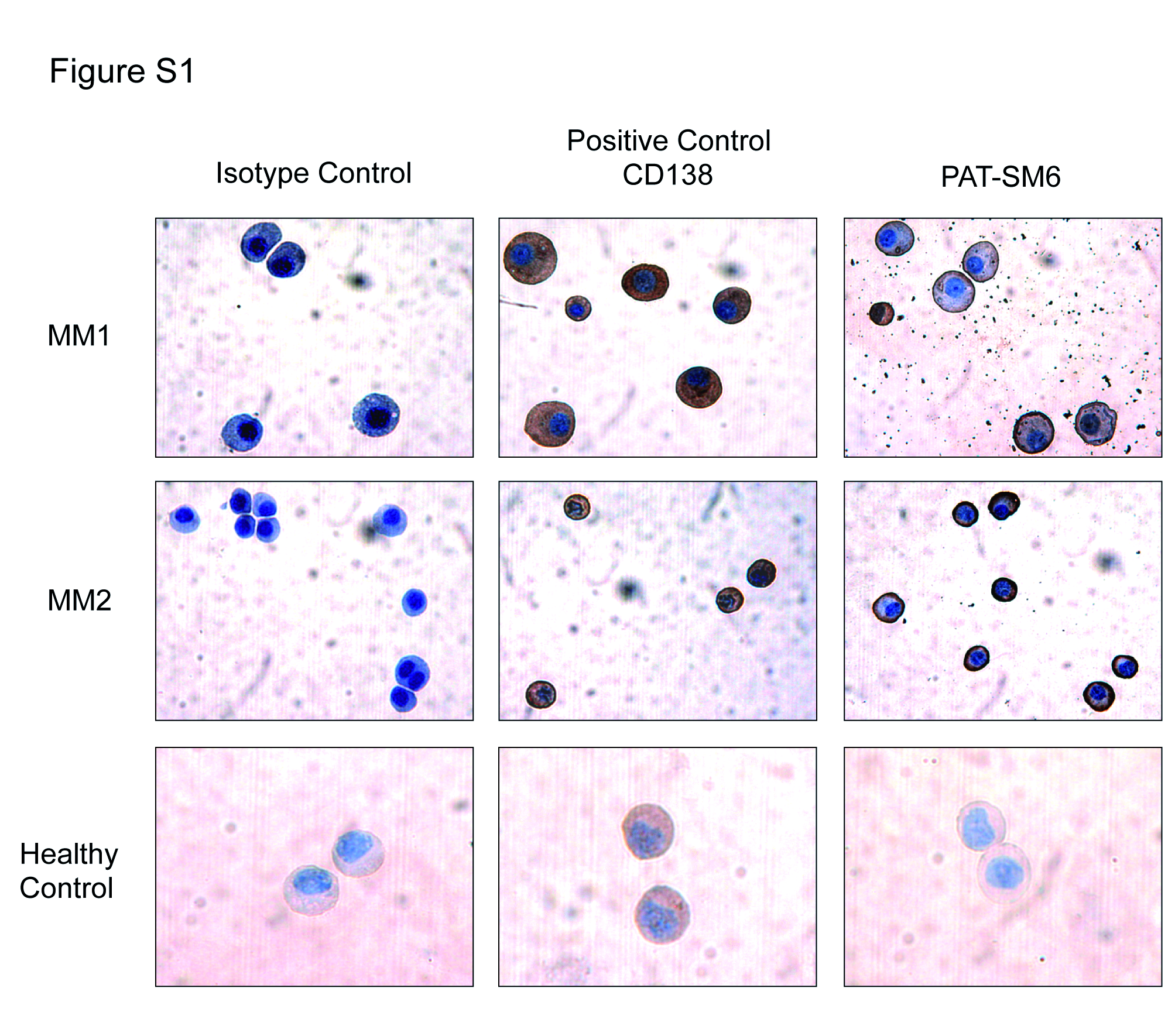

Supplement: Figure S1 — PAT-SM6 displays a membrane staining in cytospin preparations of patients MM cells. CD138-purified primary MM cells were centrifuged by cytospin, fixed and permeabilised with acetone and incubated with PAT-SM6 or controls (isotype, anti CD138). Detection followed with corresponding HRP-conjugated secondary antibodies. PAT-SM6 showed binding to CD138 purified primary MM cells specifically. As non-malignant control, plasma cells were obtained from 50 mL peripheral blood from healthy volunteers by Ficoll gradient centrifugation and subsequently CD138 isolation using magnetic beads. CD138 isolated cells from a healthy donor showed no binding when incubated with PAT-SM6. Images were captured using a Leica DM BL microscope, the Leica ICC HD digital camera and the Leica LAS EZ V2.1.0 software. (TIF) [file pone.0063414.s001.tif]

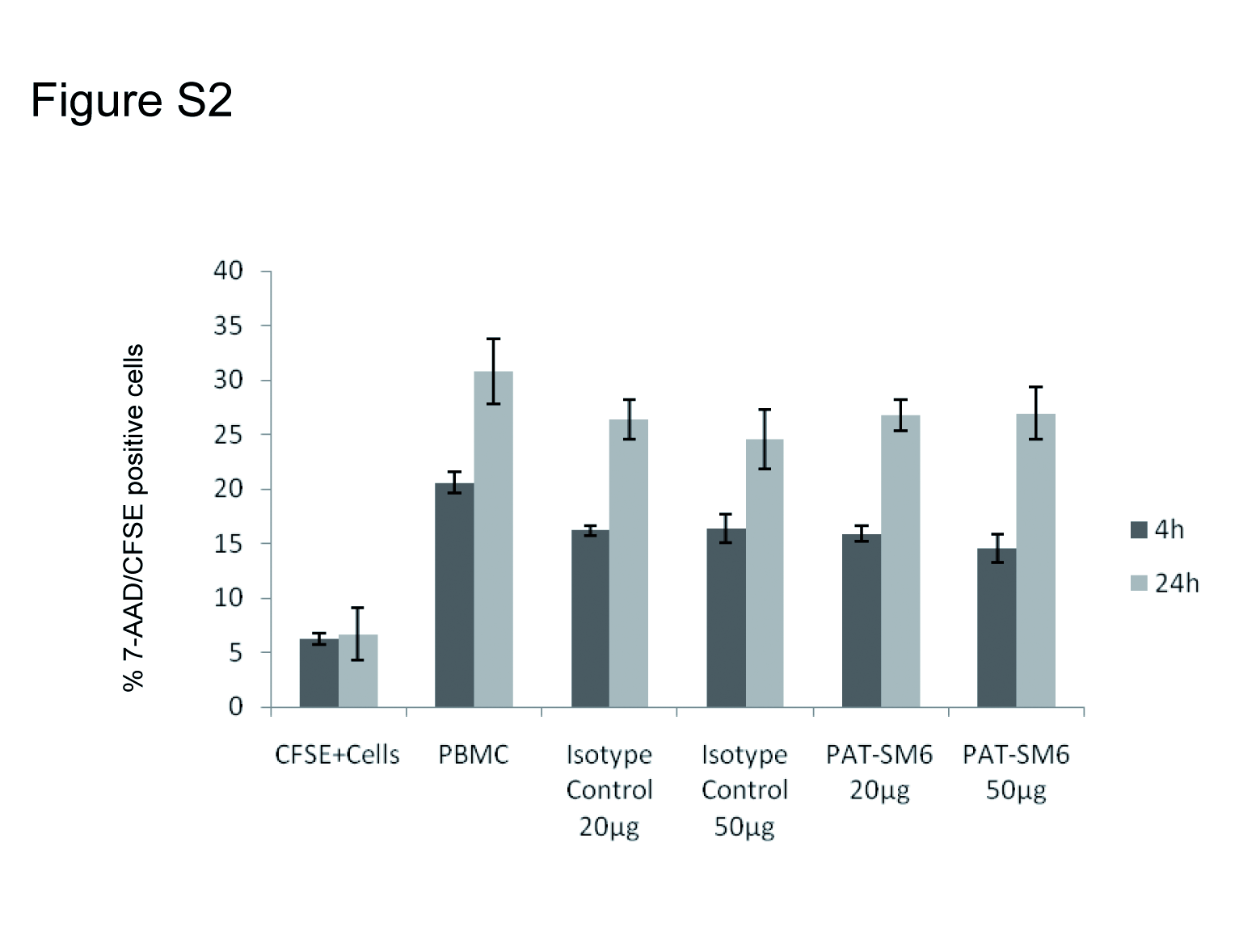

Supplement: Figure S2 — PAT-SM6 does not induce ADCC on OPM-2 cells. CFSE labelled MM cells were incubated with PAT-SM6 (25 µg/mL or 50 µg/mL) or controls (polyclonal IgM) and PBMC from healthy donors in an effector to target ratio of 25∶1 in RPMI supplemented with 1% human serum. 4 or 24 h after incubation cells were stained with 7-AAD for cell death determination and analysed by FACS. 7-AAD/CFSE double positive cells were set as MM cells killed by ADCC. (TIF) [file pone.0063414.s002.tif]

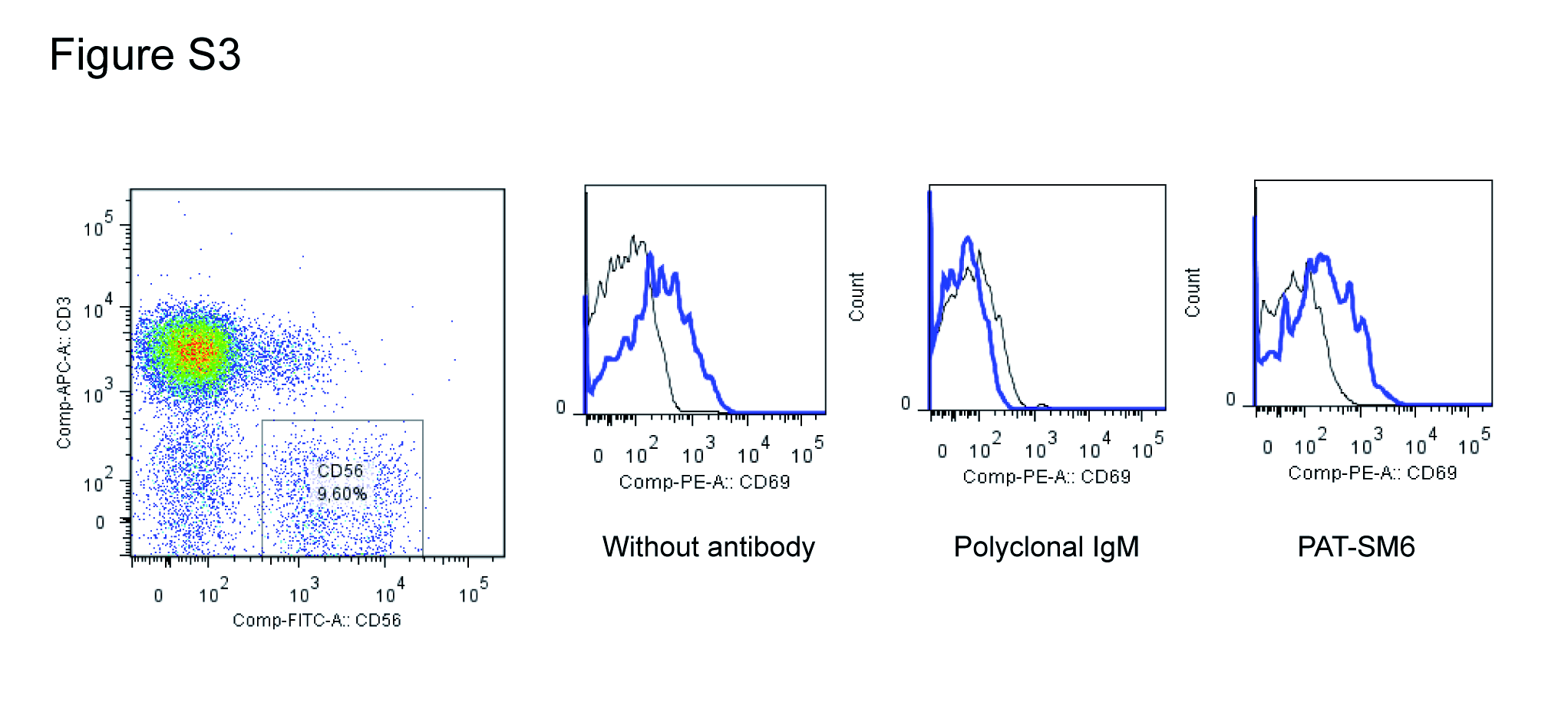

Supplement: Figure S3 — In contrast to polyclonal IgM PAT-SM6 shows no impairment in NK cell activation assessed by CD69 expression. OPM-2 myeloma cells were incubated with PAT-SM6 or unrelated polyclonal IgM (Chrompure, Dianova, Hamburg, Germany) for 15 min on ice followed by centrifugation and dismissing of supernatant to relieve unbound antibody. Cells were transferred into a 24 well plate (flat bottom) and co-incubated with PBMC from a healthy donor in an effector to target ratio of 10∶1 for 0 (black histogram) and 20 hours (blue histogram). NK cell activation was determined using CD56, CD3 and CD69 fluorescent conjugated antibodies (BD, Heidelberg, Germany). Whereas polyclonal IgM inhibits NK cell activation PAT-SM6 showed no inhibitory activity. (TIF) [file pone.0063414.s003.tif]
